# Epidermoid cyst of the coronal sulcus mimicking penile cancer: a case report

**DOI:** 10.1186/1752-1947-8-179

**Published:** 2014-06-06

**Authors:** Luigi Cormio, Vito Mancini, Francesca Sanguedolce, Paolo Massenio, Livia Fucci, Pantaleo Bufo, Antonio Traficante, Giuseppe Carrieri

**Affiliations:** 1 Department of Urology and Renal Transplantation, University of Foggia, Viale Luigi Pinto 1, 71121 Foggia, Italy; 2 Department of Pathology, University of Foggia, Viale Luigi Pinto 1, 71121 Foggia, Italy; 3 Department of Pathology, Di Venere Hospital, Piazza Ospedale di Venere 1, 70012 Bari-Carbonara, Italy; 4 Department of Urology, Di Venere Hospital, Piazza Ospedale di Venere 1, 70012 Bari-Carbonara, Italy

**Keywords:** Epidermoid cyst, Penile cancer, Surgery

## Abstract

**Introduction:**

Epidermoid cysts represent common benign tumors occurring anywhere in the body but very rarely in the penis. Only a few cases of penile localization have been reported in the literature so far, most of them being congenital and/or idiopathic, usually presenting in children as slow-growing, solitary, well-delimited cystic lesions. Here, we describe the case of a patient with a penile epidermoid cyst presenting as an ulcerated lesion of the coronal sulcus, thus mimicking penile cancer.

**Case presentation:**

A 36-year-old Caucasian man presented with a three-month history of a rapidly growing asymptomatic ulcerated lesion in the ventral portion of the penile coronal sulcus. At surgical exploration, the area under the ulcerated lesion had a well-demarcated cystic shape; following its wide excision, an intraoperative histological examination revealed an epidermoid cyst. No recurrence had occurred at nine years of follow-up.

**Conclusions:**

Rare benign tumors of the penis, like the described epidermoid cyst, may mimic cancer. Nevertheless, penile ulcerated lesions should always be surgically explored as wide excision and intraoperative histological examination remain the only means of obtaining a precise disease definition and, consequently, administering the appropriate treatment.

## Introduction

Epidermoid cysts represent common benign tumors arising from the infundibular part of hair follicles, occurring anywhere in the body but very rarely on the penis. In fact, only a few cases of penile localization have been reported in the literature so far, the vast majority being congenital and/or idiopathic lesions diagnosed during childhood [1]. Therefore, it has been suggested they develop from abnormal closure of the median raphe during embryogenesis [2,3]. Reported cases of penile epidermoid cysts presented as slow-growing, solitary, well-delimited cystic lesions with a smooth and soft appearance; when properly removed, none recurred [1,3,4].

We report here the first case, to the best of our knowledge, of a penile epidermoid cyst presenting like an ulcerated lesion of the coronal sulcus, thus mimicking penile cancer.

## Case presentation

**Figure 1:**
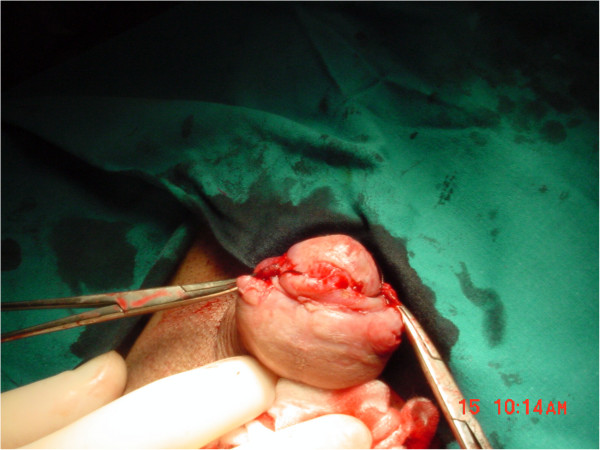
The ulcerated lesion in the ventral portion of the coronal sulcus.

**Figure 2:**
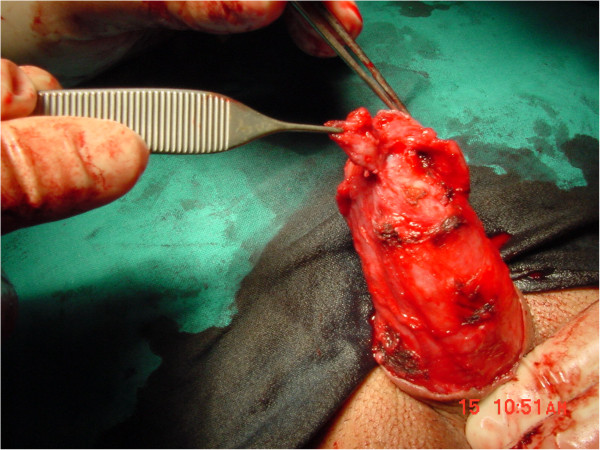
The area under the ulcerated lesion showing a well-demarcated cystic shape.

**Figure 3:**
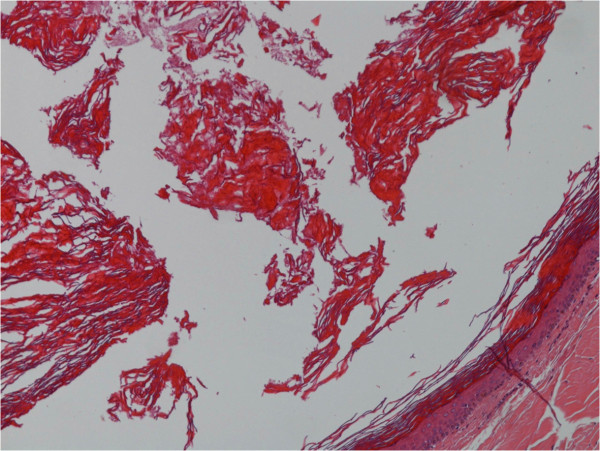
The final histological examination: stratified squamous epithelium with a keratin-filled lumen.

A 36-year-old Caucasian man presented with an asymptomatic ulcerated lesion in the ventral portion of the coronal sulcus (Figure 1), which had grown from 3 to 9mm in diameter over a three-month period. Our patient’s medical history was unremarkable; in particular, he had no history of smoking, systemic diseases, phimosis, balanoposthitis, or condilomatosis. His voiding and sexual functions were both normal. On physical examination, the lesion was a single, raised, reddish nodule, with an ulcerated surface, poorly defined borders, a hard consistency and limited mobility. There were no palpable inguinal nodes. Therefore, our patient was scheduled for surgical exploration for suspected penile cancer. We started with an incision leaving wide margins around the lesion and continuing into a circumferential incision of the inner prepuce. Once Buck’s fascia was reached, it became evident that the area under the ulcerated lesion had a well-demarcated cystic shape (Figure 2). The lesion was widely excised; an intraoperative pathology examination revealed an epidermoid cyst. The inner prepuce was then reconstructed. The postoperative course was uneventful and our patient was discharged on postoperative day 1. A final histological examination showed a cyst lined by a layer of stratified squamous epithelium with a keratin-filled lumen, thus confirming the final diagnosis of epidermoid cyst of the penis (Figure 3). No recurrence had occurred at nine years of follow-up.

## Conclusions

Epidermoid cysts of the penis are extremely rare. Most of reported cases occurred in children, thus suggesting the etiology of ‘primary’ penile epidermoid cysts being embryonic developmental defects, particularly abnormal closure of the median raphe [1]. To the best of our knowledge, there is only one case in the literature of primary penile epidermoid cyst occurring in an elderly patient [5]. Conversely, there are a few cases in the literature of ‘secondary’ penile epidermoid cysts, that is to say, they occurred after circumcision, hypospadias surgery, or penile girth enhancement surgery [6]; therefore, they are considered inclusion cysts secondary to surgical procedures that may incidentally leave islands of epithelium included in the subcutaneous tissue.

Reported cases of both primary and secondary penile epidermoid cysts presented as slow-growing, solitary, well-delimited cystic lesions with a smooth and soft appearance [1,3]. Our case is the first to present as a rapidly growing ulcerated lesion of the coronal sulcus, thus resembling penile cancer.

Almost all reported cases of penile epidermoid cyst occurred on the ventral aspect of the penile shaft, whereas only one case involved the glans penis [4]. Again, our case is the first to involve the coronal sulcus. Such a location makes the differential diagnosis with penile cancer even more difficult, as one does not expect to find a tumor normally arising from the infundibular part of hair follicles, like an epidermoid cyst, in a hairless area.

It has, however, been postulated that the coronal sulcus may act as a cleft where hairs may collect and are forced to penetrate into the penile shaft and foreskin by the natural movement that occurs between these two surfaces [7]. It could be speculated that, in our case, retention or inclusion of follicular products could have been responsible for the development of the epidermoid cyst.

Neoplastic transformation of epidermoid cysts is rare, and it has never been reported in penile cases [1-3]; in any case, a long follow-up after surgical removal is highly recommended.

In conclusion, ulcerated lesions of the penis should always be suspected of being cancer and, therefore, be surgically explored [8]. In such cases, wide excision and intraoperative histological examination remain the only means of obtaining a precise disease definition and, consequently, administering the appropriate treatment.

## Consent

Written informed consent was obtained from the patient for publication of this case report and any accompanying images. A copy of the written consent is available for review by the Editor-in-Chief of this journal.

## Competing interests

The authors declare that they have no competing interests.

## Authors’ contributions

LC conceived the study and revised the manuscript.VM drafted the manuscript. FS carried out the pathology evaluation. PM carried out the data acquisition and data analysis. FL carried out the pathology evaluation. PB participated in the pathology supervision. TA contributed to the supervision. GC contributed to the supervision. All authors read and approved the final manuscript.
